# The Latin American Integration Route and infectious diseases

**DOI:** 10.1590/1516-3180.2024.0049.R1.09092024

**Published:** 2025-04-28

**Authors:** Inara Pereira da Cunha, Sandra Maria do Valle Leone de Oliveira, James Venturini, Ruberval Franco Maciel, Antonio Jose Grande

**Affiliations:** I Escola de Saúde Pública de Mato Grosso do Sul, Campo Grande (MS), Brazil.; II Fiocruz Mato Grosso do Sul, Campo Grande (MS), Brazil; Universidade Federal de Mato Grosso do Sul, Campo Grande (MS), Brazil.; III Infectious Disease Department, Universidade Federal de Mato Grosso do Sul, Campo Grande (MS), Brazil.; IV Universidade Estadual de Mato Grosso do Sul, Campo Grande (MS), Brazil.; V Universidade Estadual de Mato Grosso do Sul, Campo Grande (MS), Brazil.

##  

An intricate relationship between growing trade, population migration, and mobility has emerged as a significant force shaping the dynamics of infectious disease spread.^1^ In today’s globalized world, the intensification of international connectivity and advancements in transportation technologies have opened unprecedented avenues for pathogens to cross borders swiftly.^2^ The recent coronavirus disease pandemic vividly illustrates how extensive transportation networks and heightened interconnectivity facilitate the rapid global dissemination of pathogens.^3^

In this scenario, the Latin American Integration Route (RILA), also known as the Bioceanic Corridor, represents a strategic logistical integration project aimed at establishing a direct connection between the Atlantic and Pacific Oceans, thus promoting efficient interconnections among different countries in South America. This ambitious corridor connecting Brazil, Paraguay, Argentina, and Chile holds the potential to boost trade, reduce distances, and strengthen economic ties among the involved nations.^4^ Its history traces back to regional integration initiatives in South America, especially among Mercosur countries, aiming to create a land Route facilitating the transportation of goods and stimulating economic development in the traversed regions.^5^

In Brazil, the RILA begins in the state of Mato Grosso do Sul and passes through strategic cities, such as Campo Grande and Porto Murtinho. Upon entering Paraguay, it traverses localities, such as Carmelo Peralta, Mariscal José Félix Estigarribia, Boquerón, and Pozo Hondo. In Argentina, the Corridor covers Misión La Paz, Tartagal, Jujuy, and Salta before reaching Chile and connecting to the ports of Mejillones and Iquique.^6^

With respect to the RILA, the state of Mato Grosso do Sul has gained attention for grappling with endemic arboviruses and infectious diseases. The intricate interplay of macro-environmental and cultural factors, involving the expansion of railway infrastructure, deforestation of natural habitats, and environmental alterations, has significantly amplified the influx of individuals, including workers, travelers, and migrants, who are eagerly pursuing economic opportunities. The establishment of settlements in close proximity to railway lines and unbridled urban expansion compounds the challenges, as evidenced by the heightened prevalence of diseases such as leishmaniasis and dengue.^7,8^ Furthermore, municipalities near the RILA in the state of Mato Grosso do Sul have shown a higher incidence of tuberculosis than the Brazilian average.^9^ This unfavorable situation may be further exacerbated by the continuous flow of people through the Bioceanic Corridor.

Directing attention to the health of travelers within this region is important and demands collaborative efforts among the nations along the RILA.^10^ The concept of “traveler health” takes center stage, emphasizing not only the well-being of individuals on the move but also the potential impact on the global public health. Educational initiatives are of utmost significance, aiming to raise awareness about the risks associated with arboviruses and emphasize preventive measures.^11^ This includes immunization as well as an understanding of the environmental factors contributing to disease transmission.^12^

Notably, since 2022, the governments of Brazil, Paraguay, Argentina, and Chile have been meeting at the “Subnational Territories Forum of the Bioceanic Capricorn Corridor” event. In 2024, the forum held its 5th meet, focusing on issues such as economic development, governance, logistics, transportation, and tourism.^13, 14, 15^ However, despite the critical health challenges posed by increased mobility, the topic of health has yet to gain significant visibility on the forum’s agenda or in international agreements. This oversight underscores the need for a greater emphasis on health strategies to address the emerging risks associated with the Bioceanic Corridor and ensure a more comprehensive approach to regional integration. The project for constructing the Bioceanic Corridor must go beyond the economic benefits,^16^ and its effects on the health and quality of life of the population should be investigated.

### Limitations

This paper offers a critical and forward-looking perspective on the health challenges associated with the RILA. However, it is important to acknowledge that this analysis is preliminary and is based on current data and scenarios that may evolve over time. The discussion presented here is limited by the data availability and the inherent complexity of the global health phenomena, which involve numerous variables and dynamic factors.

### Future directions

We have determined that health policies should be directed towards the RILA. Preventive strategies should address the root causes of disease spread, focusing on sustainable development practices that consider public health implications. Initiatives advocating responsible urbanization and reforestation play a fundamental role in mitigating the environmental factors that contribute to disease propagation. As the RILA becomes a focal point of regional integration, a comprehensive approach to global health strategies must be the public policy.

Ensuring accessible and quality healthcare services along the Corridor is the cornerstone of disease control. The strategic establishment of healthcare facilities along the RILA and ensuring the availability of necessary medical resources contribute significantly to the effective management of health risks.

Moreover, we emphasize the importance of research in understanding the intricate relationship between the RILA and health dynamics. Research initiatives should explore the specific health challenges faced by travelers along the Corridor, focusing on the prevalence of arboviruses, infectious disease transmission patterns, and socio-environmental factors influencing health outcomes. Collaborative research initiatives across the involved countries can provide valuable insights into formulating evidence-based strategies for health protection.

Formulation of cross-border agreements and protocols among the countries involved is imperative. These agreements should outline joint efforts to monitor and manage the health risks associated with the RILA. Information-exchange mechanisms, early warning systems, and coordinated responses to disease outbreaks are essential components of such agreements.

## CONCLUSION

Transnational collaboration plays a pivotal role in addressing the health challenges posed by the RILA. Comprehensive strategies encompassing education, prevention, and healthcare, supported by robust international cooperation and research endeavors, are essential to minimize the impact of population mobility on disease transmission. The conclusion of this monumental project slated for the year 2027 raises some questions. Are we truly prepared for the potential impact of RILA on Brazil and the world? Are we adequately considering the ramifications of disease spread and traveler health? The intricate interplay between increased connectivity and disease dynamics necessitates a proactive and vigilant approach to ensure the well-being of both the local and transient populations along the Corridor. As a global community, are we adept to meet this challenge and prepare for the health implications that come with such transformative endeavors?
